# Vitamin D Deficiency and Air Pollution Exacerbate COVID-19 Through Suppression of Antiviral Peptide LL37

**DOI:** 10.3389/fpubh.2020.00232

**Published:** 2020-05-28

**Authors:** Mardi A. Crane-Godreau, Kathleen J. Clem, Peter Payne, Steven Fiering

**Affiliations:** ^1^Department of Immunology and Microbiology, Geisel School of Medicine at Dartmouth, Lebanon, NH, United States; ^2^Bodymind Science, LLC, Arlington, VT, United States; ^3^Department of Medicine, Geisel School of Medicine at Dartmouth, Lebanon, NH, United States

**Keywords:** COVID-19, Vitamin D deficiency, cathelicidin/LL37, air pollution, citrullination of peptide, carbon nanoparticles, African American, tobacco smoke

## Abstract

Vitamin D deficiency and insufficiency (VDD) are widely recognized as risk factors for respiratory tract infections. Vitamin D influences expression of many genes with well-established relevance to airway infections and relevant to immune system function. Recently, VDD has been shown to be a risk factor for acquisition and severity of COVID-19. Thus, treating VDD presents a safe and inexpensive opportunity for modulating the severity of the disease. VDD is common in those over 60 years of age, many with co-morbid conditions and in people with skin pigmentation sufficient to reduce synthesis of vitamin D. Exposure to fine particulate air pollution is also associated with worse outcomes from COVID19. Vitamin D stimulates transcription of cathelicidin which is cleaved to generate LL37. LL37 is an innate antimicrobial with demonstrated activity against a wide range of microbes including envelope viruses. LL37 also modulates cytokine signaling at the site of infections. Fine particles in air pollution can interfere with LL37 destruction of viruses and may reduce effective immune signaling modulation by LL37. While vitamin D influences transcription of many immune related genes, the weakened antimicrobial response of those with VDD against SARS-CoV-2 may be in part due to reduced LL37.

**Conclusion:** Vitamin D plays an important role reducing the impact of viral lung disease processes. VDD is an acknowledged public health threat that warrants population-wide action to reduce COVID-19 morbidity and mortality. While vitamin D influences transcription of many immune related genes, the weakened antimicrobial response of those with VDD against SARS-CoV-2 may be in part due to reduced LL37. Action is needed to address COVID-19 associated risks of air pollution from industry, transportation, domestic sources and from primary and second hand tobacco smoke.

## Introduction

### Innate Immune Responses in the Context of COVID-19

Mammals have complex immune systems that integrate and coordinate adaptive and innate responses to microbial threats. Innate immune protection is the first line of defense, and is the entire defense against a novel pathogen before the slower adaptive immune system has an opportunity to respond. Humans have multiple layers of innate protection including barrier protection, cellular surveillance and communications between cells found at mucosal surfaces with other parts of the immune system. As part of this system of defense, virtually all metazoan animals, including humans, release antimicrobial peptides (AMPs) that both kill invading microbes and act as immune signaling mediators. AMPs are key element in successfully maintaining boundaries between the mammalian host and the ubiquitous microbial flora to which all life forms are exposed. An example of antimicrobial innate protection is cathelicidin (hCAP18), a broad-spectrum antimicrobial AMP known for its role in protecting against *Mycobacterium tuberculosis*, the organism that causes tuberculosis.

A cationic peptide LL-37, derived from cleavage of the cathelicidin peptide, binds to target microbes, creating a pore in vulnerable bacteria or destroying the envelope of envelope viruses such as those of the Corona virus family (1). Vitamin D (VD) activates the vitamin D receptor which is a transcription factor that influences transcription of hundreds of genes including promoting transcription of the hCAP18 gene that encodes cathelicidin. Some VD regulated genes are key to balanced responses of the immune system against many bacterial and viral infections. Recent publications (2, 3) link Vitamin D deficiency to severity of COVID-19. We postulate that with sufficient VD, that LL37 helps to clear the SARS-CoV-2 virus and helps to regulate the immune system responses. Other reports show that carbon and other nanoparticles (4) in air pollution cause citrullination of LL37 (5), which blocks its ability to destroy or disable viruses such as SARS-CoV-2.

### COVID-19 Susceptibility

A key question about COVID-19 illness is what differentiates those individuals who became seriously ill with long term health impact or death, from those who also test positive for carrying SARS-CoV-2 or having been exposed, remain symptom free or with relatively mild disease. While there are a vast array of correlations including, age, sex, ethnicity, and health status at the time of infection, most of these variables cannot be therapeutically manipulated. It makes clinical sense to identify and remediate issues that can be therapeutically adjusted, such as vitamin D sufficiency.

Appreciation of the importance of Vitamin D in the COVID-19 pandemic, requires an understanding of its role as a transcription factor for hundreds of genes, many of which are associated with immune protection (6, 7). Additionally it requires recognition that life style, geography, economics and social customs have influenced the risk of vitamin D insufficiency and deficiency (VDD) that exists in much of the world's population. We note here that vitamin D deficiency, and reduced AMPs associated with it, can be further impacted by exposure to carbon and other forms of nanoparticle-associated air pollution. Air pollution exposure is another risk factor for severe illness from COVID-19 (8).

### Vitamin D

Vitamin D is normally made by humans through exposure to adequate levels of sunlight. Broadly this means daily sun exposure to the skin for approximately 10 min. For the sun to provide adequate UVB to activate vitamin D production, the sun must be more than 45 degrees above the horizon. While the conditions for adequate UVB availability occur daily in equatorial regions of the Earth, they are only seasonally available at mid and high latitude locations. The process of acquiring Vitamin D from sunlight involves UVB converting 7-dehydrocholesterol in the skin to previtamin D_3_, and subsequently to vitamin D_3._ Vitamin D can also be obtained through some foods, generally from those that are fortified, and through supplementation (9).

Vitamin D insufficiency and deficiency are defined as follows: Vitamin D deficiency exists when 25-hydroxyvitamin D (25(OH)D) is measured at below 20 ng/ml (50 nmol/liter). Vitamin D insufficiency is defined as 25(OH)D being measured at between 21–29 ng/ml (52.5–72.5 nmol/liter) (10). VDD is found widely in industrialized societies world wide, but more so in mid and higher latitude locations as well as in older adults and in populations of color (9, 11). Relevant to the COVID-19 pandemic, extrapolating from data found at the Johns Hopkins University Corona Virus Resource Center maps showing locations and size of COVID-19 cases worldwide, to date, the greatest density of disease is occurring above 30 degrees latitude (12). Most of Europe, Asia and North America lie within this zone.

Recent news and academic reports chronicle a disproportionate percentage of people of color in the US who are hospitalized and die of COVID-19 (13–16). Extensive evidence exists that African Americans as a group, historically have significantly lower serum Vitamin D levels than Americans of European descent. This risk factor is shared, to a lesser extent by others with greater skin pigmentation and who lack adequate daily UVB sunlight exposure (17–19). VDD is shared by those whose lifestyle choices, occupation or geographic location, limit their regular exposure to sun. VDD is also widely seen in populations where religion or social custom involves wearing clothing that fully covers the body.

### Cathelicidin and LL37

Human innate immune molecule LL37, the cationic active fragment of cathelicidin (hCAP18) displays antimicrobial activity against a wide range of microbes including viral, bacterial, parasitic, and fungal microorganisms (20). The hCAP18 gene, encoding cathelicidin the precursor to LL37, is transcriptionally regulated in part by vitamin D steroid hormone metabolite, 1α,25-dihydroxyvitamin D (1,25(OH)_2_D) (21). Following cleavage of the cathelicidin peptide, LL37 is active against bacteria and viruses. Additionally, LL37 acts to modulate immune responses and functions in concert with toll-like receptors and other signaling mechanisms to communicate the nature of threat to the immune system (22–25). This nuanced modulation of the immune system serves to limit over and under responses to microbial challenges.

LL37 is reported to have attenuated the replication of a number of viruses including several classified as Class IV single stranded (SS) enveloped RNA viruses similar to the Severe Acute Respiratory Syndrome Corona Virus 2 (SARS-CoV-2) that causes COVID-19 illness. LL37 has demonstrated anti-viral activity against diverse viruses include Respiratory Syncytial Virus (RSV) (24, 26) Influenza A (27), hepatitis C (HCV) (28), Dengue virus (DENV) (29), HIV-1 (30) Vaccinia Virus (31), and others.

*In vitro* studies of cyclic mechanical stretch of human bronchial epithelial cells, show a down regulation of hCAP18 and the induction of a proinflammatory response (32). Reduction of hCAP18 means reduction of LL37. This report could have implications in terms of the decision to mechanically ventilate patients with disease symptoms similar to those found in COVID-19. Additional related studies are warranted.

### Fine Particles in Air Pollution May Interfere With Vitamin D Protection

Correlation has also been observed between exposure to higher levels of air pollution and increased levels of COVID-19 illness and deaths (8, 13).

**Figure 1:**
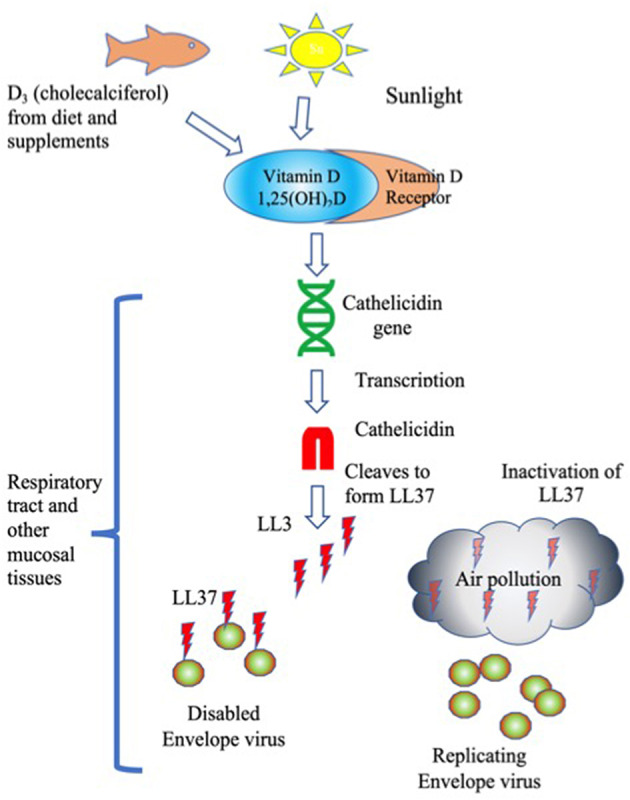
LL37 Inactivation of envelope viruses is stimulated by Vitamin D and blocked by air pollution. Humans obtain Vitamin D from sunlight, and from supplements and food. The active form of Vitamin D, 1,25(OH)_2_D binds to the Vitamin D receptor, which stimulates transcription of Cathelicidin. Cathelicidin is cleaved to generate the cationic antimicrobial peptide LL37. LL37 binds to and disables envelope viruses. Air pollution inactivates LL37 by removing the charge, leaving viruses to replicate unimpeded.

While exposure to air pollution certainly reduces lung function in multiple ways, one possibility is the impact of carbon and other types of nanoparticles (NP) found in air pollution to inactivate LL-37. NP have been shown to interfere with Vitamin D-associated innate immune protection by at least three known mechanisms, interference with antiviral activities and signaling and changes in lung tissue remodeling. See Figure 1.

Carbon NP are reported to interfere with the anti-viral actions of LL37 (33). Studies simulating cell culture exposure to industrial and transportation-associated air pollution showed that when LL37 binds to carbon NP, it is structurally altered leading to reduction of antibacterial and antiviral activities (33). Additionally, LL37 normally modulates the immune response to lipopolysaccharide (LPS) that is part of the surface of gram-negative bacteria. LL37 neutralization of the effects of LPS, as measured by decreases in TNF-alpha concentrations, is impacted by carbon NP.

The effects of fine particles in air pollution have more far reaching effects. Recent research demonstrates that LL37 can be altered by enzymatic activity of peptidyl arginine deiminases (PAD) (5). The process, called citrullination, involves changing the positively charged arginine in LL37 to citrulline and thus changing its charge from positive to neutral. This effectively removes the mechanism by which LL37 is able to destroy viruses and bacteria (5, 33). Additionally, neutralization of charge by citrullination is responsible for disabling its ability to dampen inflammatory responses to viral infections.

Air pollution from transportation and industry are high in many of the most significant COVID-19 hot spots globally (8, 13). Fine particles in air pollution that have been linked to citrullination of proteins include a variety of materials used in industry such as nickel nanoparticles (4) and carbon nanotubules (34). Exposure to primary and second hand tobacco smoke is also associated with protein citrullination (35). In addition to industrial and transportation associated air pollution, carbon nanoparticles are also generated by wood or other domestic types of fires. This may be of particular importance in areas where fires are used for cooking or heating homes.

### Vitamin D in Tissue Remodeling

Another mechanism of vitamin D protection against lung disease involves its role in balanced breakdown and repair of lung and other mucosal tissues. Primary mediators of breakdown of extracellular matrix are the matrix metalloproteinase (MMP) family of proteases, some of whose members are secreted from cells and support tissue repair and remodeling. The actions of MMPs are balanced by a family of inhibitors, tissue inhibitors of metalloproteinases (TIMP). Vitamin D has demonstrated regulatory effects on MMPs and their TIMP inhibitors (36). Possibly relevant to fine particles in air pollution, in studies of VDD mice exposed to second hand tobacco smoke, the balance between breakdown and repair is lost. This does not occur with tobacco smoke exposure or VDD alone. Under conditions of both smoke and VDD, the process is dominated by increased MMP-9 relative to its specific inhibitor, TIMP1, contributing to the breakdown of lung tissues (37).

### Bioavailability of Vitamin D

Studies involving vitamin D intervention or passive monitoring of VDD associate diseases, reportedly a positive correlation exists between either circulating levels of 25-hydroxyvitamin D (38) or a dose dependent effect of Vitamin D administration and beneficial outcomes. However, vitamin D metabolism has a variety of complex steps that modulate generation of the active 1,25-dihydroxyvitamin D (1,25(OH)_2_D) (6, 39). Complicating bioavailability, and potentially relevant to the COVID-19 pandemic, is that there are differences in Vitamin-D-binding protein in humans that are specific to populations of European vs. African ancestry (39). This is a complex topic that warrants additional attention, to understand its implications, especially with the racial differential in morbidity and mortality from COVID-19 illness.

Another issue that impacts bioavailability is dosing, specifically the benefits of daily intake of Vitamin D vs. bolus dosing. While bolus dosing studies show rapid correction of VDD, the increase in 25(OH) Vitamin D was of short duration (40–42). In contrast, daily dosing has been shown to produce sustained serum levels of 25(OH)Vitamin D (43). Measurement of Vitamin D is readily available and supplementation is inexpensive and safe if done properly. As to the role of daily vs. bolus dosing strategies, how, one, the other or a combination of approaches would impact VDD in COVID19 is unclear.

## Discussion

COVID-19 is an immediate society-altering public health crisis and understanding why severity varies from life threatening to asymptomatic is crucial to resolve this pandemic. We have postulated that vitamin D plays a pivotal role in modulating severity of COVID-19 illness; that LL37 plays a role in the clearance of the SARS-CoV-2 virus and in modulating the immune system responses; and that fine particles in air pollution may interfere with protections afforded by vitamin D and LL37. Minimally discussed to date, is strong established evidence of the importance of Vitamin D sufficiency in reducing the impact of viral lung disease processes that have implications for mitigating COVID-19. Given the relative benefits of protection afforded by attaining and maintaining Vitamin D sufficiency, it raises the potential beneficial impact of immediate attention by public health and medical providers to perform in depth studies of the relationship of Vitamin D to COVID-19 illness. Additionally, protocols for prevention, treatment and reduction of symptoms warrant immediate attention. Further, attention must be focused on the risks and long-term mitigation of exposure to fine particles from industrial and transportation associated air pollution as well as from primary and second hand tobacco smoke and from other domestic sources.

## Data Availability Statement

The original contributions presented in the study are included in the article/supplementary materials, further inquiries can be directed to the corresponding author/s.

## Author Contributions

MC-G: conception of hypothesis, manuscript, and project management. SF: manuscript and editing. PP: manuscript and editing. KC: medical expertise, manuscript, editing.

## Conflict of Interest

MC-G and PP were employed by the company Bodymind Science, LLC. The remaining authors declare that the research was conducted in the absence of any commercial or financial relationships that could be construed as a potential conflict of interest.
